# Seroprevalence of peste des petits ruminants disease in Indonesian buffaloes may be an emerging threat to small ruminants

**DOI:** 10.14202/vetworld.2024.535-539

**Published:** 2024-03-05

**Authors:** Indrawati Sendow, Heri Hoerudin, Risza Hartawan, Nuha Fairusya, Atik Ratnawati, April Hari Wardhana, Dyah Haryuningtyas Sawitri, Harimurti Nuradji, Ni Luh Putu Indi Dharmayanti, Muharam Saepulloh, Emdadul Haque Chowdhury

**Affiliations:** 1 Research Center for Veterinary Science, National Research and Innovation Agency, Cibinong, 16911, Indonesia; 2 Department of Pathology, Faculty of Veterinary Science, Bangladesh Agricultural University, Mymensingh, 2202, Bangladesh

**Keywords:** antibody, buffalo, Indonesia, peste des petit ruminants, seroprevalence

## Abstract

**Background and Aim:**

The peste des petit ruminants (PPR) is a disaster-class virus that causes catastrophic drawbacks to small ruminant industries in affected countries. As PPR disease has been reported in neighboring countries, Indonesia, which has a large population of sheep and goats, has become prone to the emerging threat of infection. Because the virus can also infect other animals with subclinical manifestations, large ruminants, such as buffaloes, may play an important role in spreading the virus in the environment. Therefore, the main objective of this study was to identify PPR seroprevalence in the buffalo population of Indonesia.

**Materials and Methods:**

A competitive enzyme-linked immunosorbent assay was performed to identify the specific antibody for PPR viruses in the buffalo population using serum bank collection from the National Research and Innovation Agency, Indonesia.

**Results:**

PPR virus seroprevalence was detected in buffalo from Central Java, East Java, and East Nusa Tenggara Province in Indonesia. Although seroprevalence was low in the population, the antibody titer was relatively high in the positive samples. Sex and age were identified as determinant factors in the seroprevalence distribution of the buffalo population.

**Conclusion:**

The presence of antibodies against the PPR virus in buffaloes may indicate that PPR virus is circulating in the buffalo population of Indonesia.

## Introduction

Peste des petits is a highly contagious viral disease caused by Morbillivirus of the Paramyxoviridae family that predominantly affects small ruminants, primarily sheep and goats [1–4]. Transmission of the disease mainly occurs by direct contact between infected and susceptible animals through aerosolized contagious particles of body fluids such as lacrimal/nasal discards, saliva, and feces [5]. With high morbidity and mortality rates, clinical disease signs are characterized by fever, conjunctivitis, stomatitis, nasal discharge, diarrhea, and bronchopneumonia [6–8]. Several studies have shown that the disease in goats is more lethal, with severe symptoms and higher mortality [6].

According to the World Organization for Animal Health (WOAH) guidelines, peste des petit ruminants (PPR) is categorized as a notifiable disease due to its significant economic repercussions on affected countries, especially in the sheep and goat farming industry [9]. PPR is a transboundary emerging disease that spreads across broad geographic areas in Asia, Africa, and the Middle East [10]. PPR has become endemic in Morocco, Tunisia, Algeria, Bangladesh, Tanzania, China, and Laos [10–15]. In 2021, the first case of PPR in Southeast Asia was recently reported to the WOAH in Thailand. As a neighboring country to Thailand, Indonesia is more prone to exotic diseases such as PPR. Although Indonesia has still been declared free from the disease, a previous study reported the presence of PPR antibodies in goat and sheep populations from the Indramayu and Solo Districts of West and Central Java Provinces, respectively [16]. This evidence suggests the presence of PPR infection in Indonesia. Subsequently, two suspected PPR cases were serologically detected in Yogyakarta Province in late March 2023, documented in a notice by the Directorate General of Livestock and Animal Health, Ministry of Agriculture of Indonesia bearing the number 24093/PW.020/F/03/2023. Therefore, it is necessary to conduct comprehensive studies with a holistic approach as early warning systems for PPR disease, which has a devastating impact on the sheep and goat industries at the national level.

The PPR virus may infect other animal species with subclinical manifestations, including large ruminants such as cattle and buffalo and wild animals such as pigs and camels [17–21]. To understand the actual disease condition in the field, the presence of the virus in these types of animals should not be ignored [20]. In addition, the previous studies have detected the presence of PPR antibodies in large ruminants, especially in buffaloes, which have a higher incidence rate than other animals [18].

Therefore, this study aimed to estimate the seroprevalence of PPR virus in the buffalo population in several areas of Indonesia, particularly by utilizing the available retrospective serum bank collection of the Research Center for Veterinary Science, National Research and Innovation Agency, Indonesia.

## Materials and Methods

### Ethical approval

The ethical clearance for this study, especially for collecting blood samples, was approved by the Ethics Committee of the Indonesian Agricultural Research and Development Agency with the approval number Balitbangtan/BBLitvet/Rm/04/2019, which originally related to a parasitic disease study in the buffalo population.

### Study period and location

This study was conducted from January 2022 to March 2023 in the Virology Laboratorium, the Research Center for Veterinary Science under the Ministry of Agriculture, Bogor, Indonesia.

### Sample origin and study area

One hundred and forty five buffalo serum samples stored in the Serum Bank collection were obtained from previous parasitic disease studies. These buffalo sera were collected from January 2017 until December 2019 from several districts in four provinces in Indonesia, including 40 from Banten, 55 from Central Java, 15 from East Java, and 35 from East Nusa Tenggara.

### Competitive enzyme-linked immunosorbent assay (c-ELISA)

Buffalo sera were analyzed for PPR antibodies using a commercial c-ELISA kit based on a recombinant nucleoprotein developed by IDvet Innovative Diagnostics, CIRAD-EMVT, Montpellier, France [16–18]. This assay was performed according to the manufacturer’s instructions. The optical density (OD) was measured at 450 nm using a Multiskan^™^ FC microplate photometer (Thermo Fisher Scientific, USA). The results were interpreted based on the percentage inhibition (PI) of monoclonal (mAb) binding in the tested serum. PI was calculated from the mean OD value using the kit’s manual instructions. Values of 50%, 50%–60%, and >60% were considered positive, ambiguous, and negative, respectively.

## Results

**Figure-1:**
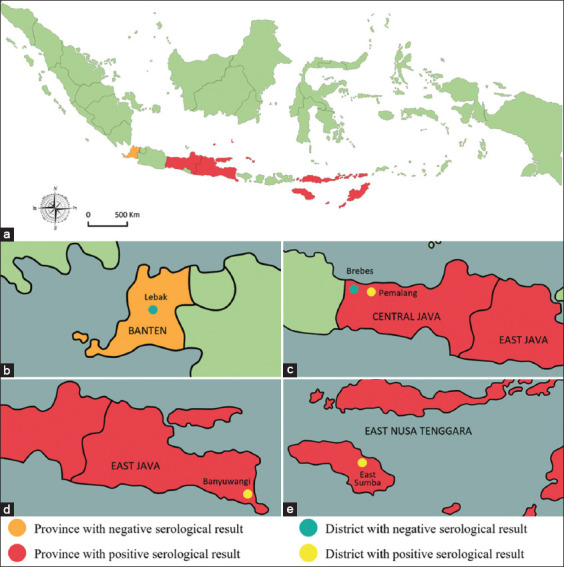
The mapping of the Peste des petit ruminants seroprevalence in buffalo population with the study areas. (a) The whole region of Indonesia, (b) Banten, (c) Central Java, (d) East Java, and (e) East Nusa Tenggara [Source: http://surl. li/qtwib] ].

A total of 145 buffalo sera from four different provinces in Indonesia were tested for the presence of the PPR virus using c-ELISA. Figure-1 depicts the mapping of PPR seroprevalence in the study area. As a result, antibodies specific to the PPR virus were identified in three provinces: Central Java, East Java, and East Nusa Tenggara province. Table-1 displays detailed information on the seroprevalence of PPR in the buffalo population of Indonesia. In this study, the overall prevalence of PPR was 4.1% across all areas. Seroprevalence was highest in sera samples from East Sumba (East Nusa Tenggara Province) at 11.4%. The PPR seroprevalence in Banyuwangi (East Java Province) and Pemalang (Central Java Province) was 6.7% and 2.7%, respectively. No PPR seropositivity was detected in the regions of Lebak (Banten Province) and Brebes (Central Java Province).

**Table-1:** The seroprevalence of the PPR antibody in the buffalo population in Indonesia.

Province	District	n	Seroprevalence (%) based on						

			Sex		Age (year)				Total
	
			Male	Female	<1	1–2	>2	Unknown	
Banten	Lebak	40	n.a.	0 (0/40)	n.a.	0 (0/3)	0 (0/37)	n.a.	0
Central Java	Brebes	18	n.a.	0 (0/18)	0 (0/5)	0 (0/5)	0 (0/8)	n.a.	0
	Pemalang	37	n.a.	2.7 (1/37)	20 (1/5)	0 (0/10)	0 (0/19)	0 (0/3)	2.7
East Java	Banyuwangi	15	0 (0/1)	7.1 (1/14)	n.a.	n.a.	6.7 (1/15)	n.a.	6.7
East Nusa Tenggara	East Sumba	35	3.3 (2/6)	6.9 (2/29)	0 (0/2)	0 (0/1)	12.5 (4/32)	n.a.	11.4
Total		154	28.6	2.9	8.3	0	4.5	0	4.1

n=Number of samples, n.a.=Not applicable, PPR=*Peste des petit ruminants*

Subsequently, PPR seroprevalence was further analyzed based on the sex and age of the buffalo. Seroprevalence was higher in males (28.6%) than in females (2.9%) according to the gender parameters. The number of females tested in the study was 19.7 times higher than that of males. On the basis of age parameters, the highest seroprevalence of PPR (8.3%) was estimated in younger buffaloes aged 1 year, followed by older buffaloes aged more than 2 years (4.5%). In addition, seroprevalence was not detected in buffaloes aged 1–2 years.

Table-2 shows the distribution of PI values of mAb specific for PPR virus at several categorical levels, especially for seropositive samples. Because ELISA is an indirect competitive platform employing a monoclonal antibody, a lower PI indicates a higher antibody titer in the serum sample against the PPR virus. Interestingly, the antibody titers on the seropositive samples from certain areas, including in Pemalang, Banyuwangi, and East Sumba Districts, are quite high, suggesting strong evidence for the PPR antibody.

**Table-2:** The distribution of PI value of the seropositive samples for the PPR in the buffalo population in Indonesia.

Province	District	n	PI value		

			50–40%	40–30%	<30%
Central Java	Pemalang	1	-	1	-
East Java	Banyuwangi	1	-	-	1
East Nusa Tenggara	East Sumba	4	-	1	3
Total		6	-	2	4

PI=The percentage of inhibition, n=Number of samples

## Discussion

It has been hypothesized that buffaloes play an essential role in the epidemiology of PPR in the environment [17, 18]. As a domesticated animal, buffaloes can be kept, grassed, and housed alongside other domestic animals, such as cattle, sheep, and goats [18]. Spillover diseases between these animals are common, especially in traditional farming practices. The buffalo may act as a reservoir and/or carrier for the PPR virus, and subclinical infections have been reported in many areas [18, 22, 23]. As expected, this study confirmed the seroprevalence of the PPR antibody in the buffalo population from 2017 to 2019 in certain regions in Indonesia, including Central Java, East Java, and East Nusa Tenggara Provinces, which have low-altitude topography between 0 and 1500 m above sea level. This evidence is consistent with other studies that identified serological evidence of PPR in large animals, such as cattle and buffaloes [18, 22]. Although no clinical signs have been observed, studies in Pakistan and Bangladesh have found higher seroprevalence in buffalo than in sheep and cattle [17, 18].

In this study, the seroprevalence of the PPR virus in the buffalo population differed according to sex and age. This study revealed a higher prevalence in males, which is consistent with the previous studies [24, 25]. However, because the proportion of males was significantly lower in this study, it may not be prudent to draw any conclusions from these results. This result may be biased because the proportion of males was significantly lower in cases where only a small proportion of adult males were kept for reproductive purposes. Conversely, several studies have found opposite results [12, 26]. In general, female animals are more susceptible to disease infection due to stress related to reproduction, such as pregnancy, milk production, and childcare [27].

The parameter of age has also previously been described as a risk factor for PPR. This study estimated higher seroprevalence in juvenile buffaloes than in older buffaloes. Several studies have reported similar and opposite outcomes [12, 26]. The higher seroprevalence in young animals may be attributable to maternal antibodies derived from parental immunity, especially after 4–6 months. Because the intensification buffalo farming system is a common practice in the area of study, juveniles could be exposed to PPR infection from infected animals, especially from pasture areas. Therefore, buffalo may have antibody titers at a young age. On the other hand, adult animals may also have higher seroprevalence due to the owner’s decision to keep female animals for a longer period of time for reproduction purposes, resulting in a longer risk period for PPR virus exposure [27].

Although the seroprevalence of PPR in buffaloes, sheep, and goats in Indonesia has been identified, there have been no reports related to PPR outbreaks in sheep or goat populations in Indonesia [16]. PPR can be falsely diagnosed because the clinical symptoms of PPR can be similar to those of other diseases such as orf, bluetongue, Mycoplasma, Pasteurella, and other bacterial infections [26]. The seroprevalence of the PPR antibody could also cross-react with other morbillivirus infections, such as rinderpest [6, 28]. Although Indonesia is free from the rinderpest virus, recent data on rinderpest infection remain unknown. Therefore, further analyses should be conducted to detect viruses in samples, for example, secretions from live animals and tissues from slaughtered animals, particularly in lymph nodes, as confirmation tests to verify the specific seroprevalence [15, 29, 30].

The results of this study should serve as a serious warning for the Indonesian government and all stakeholders to mitigate the risk of PPR outbreak. In recent years, foot-and-mouth disease infections have emerged in Indonesia [31]. The PPR virus may also bring catastrophic drawbacks to Indonesia’s sheep and goat populations. Although the transmission of the disease is limited to close contact between infected and susceptible animals, the role of other animal species as reservoirs or carriers should be considered. Therefore, the quarantine procedures of the PPR should not focus only on sheep and goats but should also apply to large ruminants and wild animals.

## Conclusion

The seroprevalence study indicates that the buffalo population in Indonesia is in contact with the PPR virus, and therefore, there may be a possibility of spillover of the virus in the susceptible populations. A continuous surveillance program is required to monitor the susceptible population to prevent disease outbreaks.

## Recommendation

Active surveillance of ruminants for the serological and molecular detection of the PPR virus should be implemented. Samples should be used from the resident ruminants of hot spots, particularly from the border area where animal trespasses usually occur. Several scenarios in disease surveillance, such as more sensitive tests, increasing sample size, and widening sampling locations as well as the number of animal species, including wild animals, should be implemented to detect the virus’s existence.

## Authors’ Contributions

IS, AR, HH, AHW, and DHS: Supervised the field sampling, data collection, and laboratory work. IS, RH, AHW, and EHC: Data entry, analysis, and interpretation, and participated in the preparation of the manuscript. IS, AR, RH, NF, HN, NLPID, MS, and EHC: Conceptualized and designed the study and reviewed and edited the manuscript. All authors have read, reviewed, and approved the final version of the manuscript.
